# Natural Products as a Potential Source of Promising Therapeutics for COVID-19 and Viral Diseases

**DOI:** 10.1155/2023/5525165

**Published:** 2023-04-15

**Authors:** Soheila Bafandeh, Ehsaneh Khodadadi, Khudaverdi Ganbarov, Mohammad Asgharzadeh, Şükran Köse, Hossein Samadi Kafil

**Affiliations:** ^1^Research Center for Pharmaceutical Nanotechnology, Tabriz University of Medical Sciences, Tabriz, Iran; ^2^Department of Chemistry and Biochemistry, University of Arkansas, Fayetteville, AR 72701, USA; ^3^Research Laboratory of Microbiology and Virology, Baku State University, Baku, Azerbaijan; ^4^Biotechnology Research Center, Tabriz University of Medical Sciences, Tabriz, Iran; ^5^Department of Infectious Diseases and Clinical Microbiology, Dokuz Eylül Üniversitesi, Izmir, Turkey; ^6^Drug Applied Research Center, Tabriz University of Medical Sciences, Tabriz, Iran

## Abstract

**Background:**

A global pandemic has recently been observed due to the new coronavirus disease, caused by SARS-CoV-2. Since there are currently no antiviral medicines to combat the highly contagious and lethal COVID-19 infection, identifying natural sources that can either be viricidal or boost the immune system and aid in the fight against the disease can be an essential therapeutic support.

**Methods:**

This review was conducted based on published papers related to the herbal therapy of COVID-19 by search on databases including PubMed and Scopus with herbal, COVID-19, SARS-CoV-2, and therapy keywords.

**Results:**

To combat this condition, people may benefit from the therapeutic properties of medicinal plants, such as increasing their immune system or providing an antiviral impact. As a result, SARS-CoV-2 infection death rates can be reduced. Various traditional medicinal plants and their bioactive components, such as COVID-19, are summarized in this article to assist in gathering and debating techniques for combating microbial diseases in general and boosting our immune system in particular.

**Conclusion:**

The immune system benefits from natural products and many of these play a role in activating antibody creation, maturation of immune cells, and stimulation of innate and adaptive immune responses. The lack of particular antivirals for SARS-CoV-2 means that apitherapy might be a viable option for reducing the hazards associated with COVID-19 in the absence of specific antivirals.

## 1. Introduction

COVID-19, an infectious illness produced by SARS-CoV-2 (severe acute respiratory syndrome coronavirus-2), affects the lower respiratory tract and the hematological system. Its key clinical manifestations, such as fever, cough, and shortness of breath, are comparable to other types of viral pneumonia [1]. There are several ways that COVID-19 can spread but coughing or sneezing droplets is the most common [2]. Some preventive measures, such as social distancing and lockdown of cities, have been adopted to control the COVID-19 pandemic [3]. SARS-CoV-2 has become a global public health hazard despite concerted measures to maintain the virus's high transmissibility in specific places [4, 5]. At a minimum, an optimal therapy should speed symptomatic recovery, limit viral transmission in the population through early viral clearance from the infected individuals, and reduce mortality [6]. We have a long way to go before we have a medication for severe COVID-19 patients that is successful though.

New viral-born infectious diseases have challenged the life of humans and other living creatures. Usually, viral diseases are difficult to control and have a more comprehensive public health impact, so viral pathogens have received more attention and have threatened modern healthcare and pharmaceutical sectors. Preventive and supportive treatments are now used to avoid future problems and organ damage from COVID-19. Due to the public health issue produced by COVID-19, researchers have concentrated on identifying medicines with therapeutic or preventive potential for the treatment and control of this viral illness [7–9].

Four principal viral structural proteins are spike (S), envelope (E), membrane (M), and nucleocapsid (N) [10, 11]. SARS-CoV-2 (ACE-2) receptors, RNA-dependent RNA polymerase (RdRP), and spike proteins are critical sections of novel therapeutic targets based on the current knowledge [12, 13]. The antiviral mechanism for plant extracts differs as per the structure and the replication process of the viruses; some plants can help boost our body's natural antiviral immunity [14, 15]. Peptides and proteins of medicinal significance can be synthesized from plant extracts, leading to vaccinations and protein/peptide-based treatments [16, 17]. Meanwhile, testing new therapeutic items will take a long time, and many ethnic groups have already examined medicinal plants since ancient times. Nearly 80% of the world's population relies on medicinal plants as their major source of healthcare [18]. Moreover, plant- and microbial-based natural compounds make up more than 40% of the synthetic drugs on the market [19]. Researchers should focus on the screening of hundreds of natural products to locate the powerful antiviral medicine for COVID-19. As a result, the focus of this study is on medicinal plants and herbs that display antiviral activities and might be valuable in drug discovery efforts.

## 2. Medicinal Plant Candidates

High costs and adverse effects of synthetic drugs and the arrival of adverse drug reactions (ADRs) have required harmless and unique antiviral drugs [20]. Herbal essences have become an outstanding choice for the formulation of antiviral medicines that can hinder multiple steps of the virus replication cycle [21]. According to the features of the SARS-CoV-2, a molecular mechanism of the host is associated with the immune response [22]. In this situation, in which the preventive and therapeutic representatives have not been developed and advised for patient administration, herbal medicines are frequently used by many individuals in the community (Table 1).

### 2.1. *Salvia miltiorrhiza*

**Figure 1:**
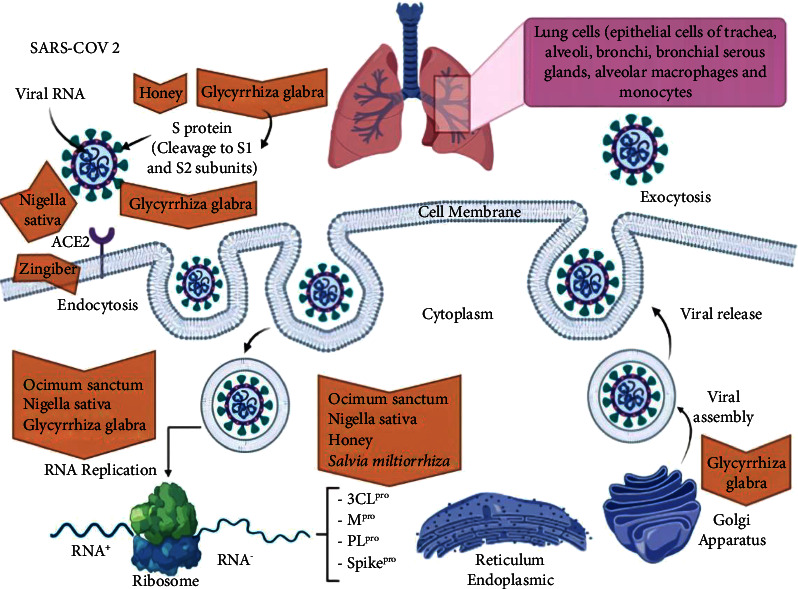
The schematic mechanism of SARS-CoV-2 entry and replication into the host cell. S (spike) protein on the surface of SARS-CoV-2 binds to the ACE2 receptor on the surface of the host cell and enters it by endocytosis. Then, S protein is cleaved to S1 and S2 subunits. The viral genome is then released into the cytoplasm and translated through the ribosome. In the following, negative sense RNAs are produced from positive sense genomic RNA of virus as a template. Later, some structural and nonstructural proteins are also manufactured, e.g., M^Pro^, 3CL^pro^, PL^pro^, and S protein. The viral proteins and genome RNA are assembled in the ER and Golgi apparatus compartment and then inserted into the vesicles. Vesicles containing the virus, transported to the cell membrane and release via exocytosis. At each stage, natural products' inhibitory effects on coronavirus pathogenicity are indicated by a red arrow. The virus may also leave the cell by budding. The figure is provided by BioRender.

The underlying antiviral mechanisms can be divided into two categories: the direct inhibition of viruses and the indirect antiviral effect. *Salvia miltiorrhiza* works through a second process that inhibits the inflammatory response mediated by the virus by modulating the function of the immune system [48]. On the other hand, some main protease enzymes are important in virus replication, such as 3C like protease (3CL^pro^) and papain-like protease (PL^pro^) [23]. One of *Salvia miltiorrhiza's* most robust and most effective PL^pro^ inhibiting compounds is tanshinone, a diterpene with the structure of abietane. Moreover, this inhibitor can also act against 3CL^pro^. Tanshinones' biological activities are anti-inflammatory activity, cardiovascular effects, and antitumor activity. Depending on the type of the enzyme (3CL^pro^ or PL^pro^), tanshinones have different selective inhibitory activity against these SARS-CoV enzymes (Figure 1). The inhibitory effect of different tanshinones against PL^pro^ has been well proven [24, 49].

Ethanolic extract of *Salvia miltiorrhiza Bunge* has the most inhibition effect on PL^pro^ of SARS-CoV. In addition, seven bioactive tanshinones of n-hexane fraction of the ethanolic extract (tanshinone IIA, tanshinone IIB, methyl tanshinonate, cryptotanshinone, tanshinone I, dihydrotanshinone I, and rosmariquinone) can inhibit the PL^pro^ activity of SARS-CoV. Cryptotanshinone was the most potent inhibitor of SARS-CoV PL^pro^ [24]. *Salvia miltiorrhiza* can inhibit inflammatory response through its well-known flavonoid tanshinone IIA (Tan IIA) [50]. Moreover, the suppression of inflammation and disruption of the inflammatory signaling cascade in both the mice cardiac tissue and H9c2 cells is caused by pretreatment of Tan IIA through the nuclear accumulation of nuclear factor erythroid 2-related factor 2 (nrf2), triggering the expression of its downstream gene hemeoxygenase-1 (HO-1) and NADPH dehydrogenase quinone-1 (NQO-1) and then the effect of Forsythiae Fructus water extract on Nrf2/HO-1 signaling [51–53]. Activities of SOD (superoxide dismutase), catalase (CAT), and GSH-Px in serum can be increased, and the generation of ROS in doxorubicin(DOX) can induce cardiotixicity in animals.. Effective chemicals can decrease hyperlipidemia in rats *in Salvia miltiorrhiza* [25].

### 2.2. Glycyrrhizin

It is a triterpenoid saponin Glycyrrhizae Radix (GLR) obtained mainly from the roots of the *Glycyrrhiza glabra* plant [54]. Glycyrrhizin's many pharmacological characteristics, including anti-inflammatory, antioxidant, antiallergenic, antibacterial, antiviral, antiparasite, and anticancer capabilities, have been intensively explored in biology and medicine [55–57]. Glycyrrhizin is a multitarget substance whose potential undiscovered targets are revealed over time. It has been disclosed that diammonium glycyrrhizin treatment helped severe COVID-19 [58].

Glycyrrhetinic acid (GA), a sapogenin moiety, can be glycosylated to GLR and the two glucuronic acids [26]. GLR is administered both orally and intravenously in humans. Oral administration of GLR is metabolized to GA by intestinal bacteria and absorbed through the intestine, and it can then be found in human plasma [59]. GLR's impacts on various human viruses have been examined over several years. *Glycyrrhiza glabra* (Leguminosae family) exhibits an antiviral activity against several viruses, including cytomegalovirus, herpes simplex type-1, hepatitis A, B, C, varicella-zoster, and HIV [60–62].

GLR inhibited virus replication estimated by drug-induced nitrous oxide synthase production and adsorption and penetration of the virus into cells [63]. GLR acts by inhibiting virus plasma membrane penetration (membrane effect) and suppression of viral antigen secretion (cytoplasmic effect) in hepatitis A and B viruses, respectively [27]. GLR disrupts the antigen sialylation and intracellular transmission also causes diminishing of motion of molecules in the membrane and prevents pore formation for virus entry by its saponins [58]. Because GLR is a nonlytic saponin and does not affect the integrity of the whole membrane, it has a modest permeabilizing and hemolyzing effect and a low rate of liposome leakage [64]. Glycyrrhizin has been shown in clinical research to have possible inhibitory action in replicating two clinical isolates of coronavirus (FFM-1 and FFM-2). It has been suggested to evaluate for SARS treatment [63].

In addition, rod-like micelle and fibril formation of GLR is due to its amphiphilic and anisotropic structure and self-assembling into the fibrillary network [65]. GLR possesses two advantages: first, it can boost the solubility of poorly soluble drugs; and second, it can raise these drugs' passive diffusion through cell membranes. Different drug delivery systems are embedded for certain diseases, including hepatocellular carcinoma by GLR [66].

Cholesterol is an essential compound in lipid membranes. One of the interactions of GLR is with membrane cholesterol which can cause membrane disorganization and disturbance and act by improving ions and small molecules' permeability to the membrane [58]. In the raft monolayer model, GA can perform even better than GLR. This disorganization is a critical point in surface attachment by the ganglioside-binding domain of the spike (S) protein of SARS-CoV-2 to the respiratory cells. Additionally, it has been presented that GLR can reduce the quantity of cholesterol in lipid rafts and prevent the translocation of TLR-4 to lipid rafts [67].

Combining of some antiviral drugs such as chloroquine and hydroxychloroquine with GLR can weaken virus entry to the host cell [68, 69]. Lately, it has been suggested that SARS-CoV-2 infection can be reduced by inhibiting the viral lipid-dependent attachment of the novel coronavirus to the host cell's plasma membrane by natural products like sterols and cyclodextrin [70]. Furthermore, lipid rafts are crucial in SARS-coronavirus infection because they can be an entry port for the abovementioned virus. Due to decreased viral particle formation, cholesterol depletion with methyl-cyclodextrin can diminish coronavirus infection [69, 71]. Hence, impediments of lipid rafts and cellular cholesterol metabolisms that play an essential role in the entry of viruses and their infectivity can be considered a principle.

GLR has anti-inflammatory and immune modulator features through several pathways such as toll-like receptor signaling and MAPK. Strong binding of GLR to HMGB1 distributed the protein interactions such as advanced glycation end products (RAGE), TLR2, and TLR4. The anti-inflammatory effects of GLR via TLR4/HMGB1-dependent are well proven [30]. The reduced TLR activity is associated with the diminished inflammatory cytokine and inflammatory mediator activity, such as the TLR4 ligand nicotinate phosphoribosyltransferase. It has been demonstrated that GLR can bind to more proteins like serum albumin and bind to nucleic acids, DNA, and RNA even weakly [29]. GLR has been revealed to disturb the autophagy process in infected host cells, so this point can be beneficial [72]. A study suggested that GLR could be used alongside some drugs such as chloroquine to promote solubilization and bioavailability of drugs, virus replication inhibition, and complement the drug activity, which can have synergistic effects in some circumstances [73].

Among the other 44 compounds, glycyrrhizic acid derived from *Glycyrrhiza uralensis* Fisch was the best option for SARS-CoV-2 S1 subunit attachment. Disturbing of receptor-binding domains (RBDs) of SARS-CoV-2 and angiotensin-converting enzyme II (ACE2) interaction by binding to S1 protein could be a potential target of glycyrrhizic acid even at low concentrations (IC50 = 22 *μ*M). According to the molecular docking results, two strong hydrogen interactions with Asp405 and Arg408 in the carboxyl of ring E, a strong hydrogen interaction with ARg403 in the carbonyl of ring C, plus a strong hydrogen interaction with Tyr453 in glycosyl, show that glycyrrhizic acid could be a multitarget inhibitor and a potential candidate for SARS-CoV-2 infection treatment. Glycyrrhizic acid has modest cell toxicities to transfected HEK293 cells, mouse aorta smooth muscle cells (MASMCs) even at high concentrations (100 *μ*M), and also human lung cells [28].

### 2.3. *Vitis vinifera*

Grapes like *Vitis vinifera* have long been known for their nutritional and therapeutic benefits. Glucose, organic acids, and polyphenols such as flavonoids (quercetin), tannins, and stilbenes are all present in this medicinal fruit (resveratrol and viniferins) [74]. Resveratrol represents a wide range of pharmacological and therapeutic activities such as anti-inflammatory, neuroprotective, cardioprotective, hepatoprotective, and antibacterial effects [75]. In vitro, resveratrol shows both the inhibition of infection and a decrease in MERS-CoV replication [42]. This means that resveratrol's anti-MERS and anti-SARS-CoV2 properties can be explored further. It has been revealed that the antioxidant polyphenol resveratrol protects against free radical damage in disorders such as cancer, diabetes, heart disease, neurological disease, and microbial infection [76, 77].

Resveratrol enhances resveratrol by decreasing the phosphoinositide 3-kinase/A serine/threonine protein kinase (Akt)/mTOR signaling pathway and increasing AMPK and SIRT1 pathways autophagy and killing cancer cells [78]. As an antiviral agent, resveratrol is effective against a wide range of viruses, including the herpes simplex virus, enterovirus 71, the Epstein–Barr virus, the respiratory syncytial virus, influenza, and the Middle East respiratory syndrome-coronavirus, a relative of the SARS-CoV-2 virus that causes MERS [42, 79]. SARS-CoV-2 replication and cytokine storms may be reduced if copper and resveratrol are administered together [43].

### 2.4. *Zingiber*

Ginger is the root of the *Zingiber officinale* plant, which is a member of the Zingiberaceae family. It is one of the most widely used spices with therapeutic characteristics [80]. *Zingiber* contains a group of polyphenols known as diarylheptanoids, which have been shown to have anti-inflammatory properties [81]. It can facilitate the immune response and is beneficial for COVID-19 prevention. There are some instances in which the severity of COVID-19 is attributed to macrophage hyperinflammation, even though COVID-19 is not a disease of inflammation by itself [44].

In certain circumstances, ginger's anti-inflammatory properties could help reduce symptoms and illness severity. As a result, ginger has been demonstrated to have therapeutic effects on metabolic illnesses such as diabetes and cardiovascular disease in animal models [82]. Diabetes and cardiovascular disease have a higher death rate in persons who test positive for COVID-19; therefore, this is an important consideration [83]. As a result, ginger's antiviral properties also have antioxidative, immunomodulatory, and anti-inflammatory properties [84]. The presence of allicin in ginger is reported to have anti-influenza cytokines, an effective traditional remedy against common cold viruses [85]. COVID-19 virus has been demonstrated to be an efficient antiviral because 6-gingerol has a high affinity for various binding sites on viral protein molecules [86]. Thus, ginger, with its long history of use in traditional medicine for the treatment of infectious diseases, has become a promising source of antimicrobial agents.

### 2.5. Curcuma

For many years, *Curcuma* has been a commonly used yellow spice with medicinal properties like ginger. Curcumin is also a source of diarylheptanoid polyphenols, as previously indicated [87, 88]. Curcumin is one of the other diarylheptanoids from *Curcuma longa* that represents a good inhibitory activity against PL^pro^ and has various therapeutic properties like antihyperlipidemic, anti-inflammatory, and antimicrobial activities [45]. In combat against COVID-19, nutritional supplementation is recommended to bolster the immune system, and curcumin may be a good choice for this purpose [89]. Curcumin's therapeutic effects have been studied extensively. Its antiviral activity has been observed against a variety of viruses, including emerging arboviruses like Zika virus (ZIKV) or chikungunya virus (CHIKV), hepatitis viruses, respiratory influenza virus, herpes simplex virus-2, papillomavirus, and human immunodeficiency virus (HIV) [90].

As an antiviral agent, curcumin can exert its effects in various ways, including via disrupting viral pathways or cellular processes or directly on virus-encoded proteins [91]. One recent study found that curcumin-derived carbon quantum dots could enhance curcumin's antiviral activities *in vitro* and *in vivo* against enterovirus 71 (EV71) through various pathways [92]. The entry receptor of HCoV-229E was discovered to be inhibited by carbon quantum dots alone, making them efficient against the human coronas virus (HCoV) [93]. Various intracellular small oxidative compounds may be scavenged by curcumin's ability to transport electrons [94]. Anaerobic fermentation is exacerbated, and the energy supply is reduced in severe COVID-19 cases because of pneumonia, which interferes with cell metabolism [95]. Curcumin, a powerful antioxidant, has been found to enhance the production of antioxidant enzymes and neutralize free radicals [96]. Acute sepsis-induced lung damage in rats is accompanied by an increase in the activity of superoxidase dismutase (SOD) and recovery of the levels of xanthine oxidase (XO) and total antioxidative capacity (TAOC). In contrast, MDA levels are reduced [97]. Curcumin has been shown to have antioxidant, anti-SARS-CoV-2, and perhaps immune-enhancing properties. There is a possibility that curcumin could play a role in the prevention and control of COVID-19.

### 2.6. Honey

For many years, honey has been used for medicinal purposes such as wound healing, antimicrobial, antiviral, immune booster, anti-inflammatory, antifungal, antioxidant, antidiabetic, cardioprotective, neuroprotective, antimutagenic, and antitumoral [98]. Honey has been shown to cure various viral respiratory disorders, like pneumonia, throat infection, and bronchitis; thus, it might relieve pneumonia caused by coronavirus [99]. Hydrogen peroxide (H_2_O_2_) is an antibacterial component found in honey [31]. Combining honey with some nutrients such as cinnamon, garlic, and ginger increases its antimicrobial and immune booster effect [100]. As abovementioned, honey possesses antiviral activities that can act against some viruses such as HIV, varicella-zoster virus (VZV), herpes simplex virus (HSV), respiratory syncytial virus (RSV), influenza viruses, and adenovirus as a result of low pH, osmotic effect, and some natural compounds such as lysozyme, flavonoids, hydrogen peroxide, and phenolic acids [98].

There are two fundamental ways that honey can show its antiviral effect. One way is the nitric oxide (NO) pathway by raising NO as a principal cellular neurotransmitter in multiple physiological procedures [101]. The second way also has two parts. First, it is attributed to honey's fatty acid 10-hydroxy-2-decenoic acid (10-HAD). In this way, it has been suggested that honey acts by eradicating the virus by leukocyte adhesion to the virus via 10-HAD induction. Second, strength in antiviral immunity is due to promoting the maturation of dendritic cells (DCs) derived from human monocytes and the capability of T helper cell type-1 (Th1) polarization by 10-HAD [32]. Medicinal effects of honey such as antiviral, neuroprotective, and antioxidant are due to its small components, including phenolic acids, phenols, flavonoids, carotenoids, and terpenes [102].

The antiviral activity of flavonoids in honey and propolis like quercetin and its derivatives (e.g., isorhamnetin, isoquercetin, quercitrin, and rutin) has represented against human respiratory syncytial, human metapneumovirus, influenza virus, human rhinovirus, parainfluenza, and betacoronavirus (SARS-CoV) through the critical viral enzyme, 3-chymotrypsin-like cysteine protease (3C-likepro) of SARS-CoV inhibition, as a promising target in coronaviruses [103]. A potential action of honey against 3C-like pro of SARS-CoV-2 is attributed to six compounds, including galangin, lumichrome, caffeic acid, 3-phenyllactic acid, phenethyl ester [CAPE], and chrysin, which was found by molecular modeling [33]. Synergistically, honey presents more efficacies with antibiotics. Honey ameliorates the innate immune system and stimulates the adaptive immune system, especially in upper respiratory tract infections, with its polyphenolic compounds [34]. Some studies have suggested that a component of honey called methylglyoxal may have an antimicrobial activity, but this has not been explained in detail [104].

In honey, the richest phenolic acids are vanillic acid, p-hydroxybenzoic acid, caffeic acid, gallic acid, p-coumaric acid, syringic acid, and chlorogenic acid [105]. Moreover, most flavonoids include chrysin, galangin, pinobanksin, apigenin, luteolin, pinocembrin, quercetin, genistein, and kaempferol [106]. It has been revealed that the bioactive compounds of honey and propolis, including ellagic acid, hesperetin, kaempferol, artepillin C and p-coumaric acid, and quercetin, were the most promising compounds on COVID-19 RdRp and Mpro [107]. Among them, quercetin is the most active one on the Mpro in micromolar doses. It has been proven against 3CLpro and PL^pro^ of SARS-CoV and 3CLpro protease of Middle Eastern respiratory syndrome coronavirus (MERS-CoV) [108].

As an immune booster, honey can be used as a supportive treatment for patients infected with novel coronavirus and as preventive ways in healthy people [109]. Hence, taken from the reports, three effects from honey, inducing lymphocyte proliferation and activation, inhibiting the production of proinflammatory cytokines, and inducing autophagy machinery, have been expected. It should be noted that lymphocytopenia is one of the immune problems caused by COVID-19. SARS-CoV-2 causes excessive inflammatory responses by fusing the membrane through the S protein and infecting T-lymphocytes. This process is attributed to COVID-19 mortality due to lymphocytopenia; nevertheless, SARS-CoV-2 can proliferate infected T lymphocytes.

### 2.7. *Nigella sativa*

*Nigella sativa* is a tiny black seed taken from a flower in the Ranunculaceae family and utilized as a medicinal substance for many years for various diseases. This plant is commonly grown in the Middle East, Europe, and Asia. It is also known as black cumin or black seed [110]. Antiviral, antibacterial, anti-inflammatory, antidiarrheal, and antitussive properties and many other medicinal properties have been found in *N. sativa*. *N. Sativa* has also been an antioxidant, immunomodulatory, diuretic, liver tonic, and digestive stimulant [111]. It is effective against a wide range of infectious and chronic noninfectious diseases such as diabetes mellitus, dyslipidemia, hypertension, neurologic disorders, inflammatory disorders, cancer, asthma, bronchial headache, gastrointestinal problems, and dysentery [40, 112]. Its antioxidant activity may help reduce the oxidative damage of organs caused by the virus [113]. In addition, some other components such as minerals (sodium, potassium, iron, calcium, copper, magnesium, and phosphorus) and vitamins (vitamin E, riboflavin, niacin, folic acid, pyridoxine, and thiamine), essential amino acids, carbohydrates, proteins, and fats can be found in *N. sativa* [35, 114].

*N. sativa* can be used as an adjuvant in patients infected with novel coronavirus at 40–80 mg/kg/day doses as oil in combination with drugs used to treat coronavirus without any side effects to ameliorate patients [36]. Moreover, in some studies, it has been suggested that *N. sativa* shows not only antihistaminic results due to inhibiting the release of histamines and leukotrienes and blocking histamine receptors but also represents an anti-inflammatory activity by the inhibition of nuclear factor kappa B (NF-*κ*B) [38, 40, 115]. The immunomodulatory property of *N. sativa* is related to some of its bioactive compounds, which boost immunity by increasing T lymphocytes and natural killer cells to overcome the symptoms associated with COVID-19, including inflammation and oxidative stress [116].

*N. sativa* contains bioactive compounds including terpenes (thymoquinone (TQ), dithymoquinone (DTQ), carvone, limonene, trans-anethole, and p-cymene), phytosterols, coumarins, flavonoids, saponins, isoquinoline alkaloids (nigellicimine, nigellicimine-N-oxide, and *α*-hederin), indazole alkaloids (nigellidine and nigellicine), cardiac glycosides, tannins, fatty acids, volatile oils, and phenolic compounds detected by phytochemical screening [37]. Elevated serum interferon-gamma levels, increased CD4 counts, enhanced suppressor function, and increased macrophage counts result from *N. sativa* antiviral properties [117].

On the other hand, *N. sativa* possesses some components that bind to specific targets, including some proteins and pathways such as chemokine cancer, relaxin signaling pathway, PI3K-Akt signaling pathway, IL-17, HIF-1 signaling pathway, AGE-RAGE, VEGF pathway, FoxO pathway, nuclear receptors, cytochrome P450, oxidoreductases, erasers, lyases, enzymes, family A G protein-coupled receptors, calcium signaling pathways, and circadian pathways to represent its protection activity [118]. *N. sativa* seeds contain a wide range of immune-stimulating, antibacterial, and anti-inflammatory compounds, including unsaturated fatty acids, saponins (melanin), fixed oil classes, essential oil, alkaloids, and proteins [119]. In a clinical trial, oral *N. sativa* oil in doses of up to 5 g per day for 12 weeks has shown safe results [120].

The inhibition property of SARS-CoV-2 is related to its active constituents, including *α*-hederin and nigellidine [121]. A-hederin is a saponin that exhibits various anti-inflammatory, antioxidant, antitumor, antifungal, and antiparasite activities and has shown impressive effects on asthma and cancers in vivo and acts better than chloroquine, hydroxychloroquine, and favipiravir [38]. Hederagenin is another saponin that is present in *N. sativa*. Nigellidine is a significant alkaloid that acts like chloroquine and is better than hydroxychloroquine and favipiravir [122]. Nigellidine represents relatively good binding affinity to some proteins and enzymes of SARS-CoV-2, including spike-glycoprotein, nonstructural protein 2, N-terminus-protenase, nucleocapsid, and 6LU7 [41]. Moreover, it shows high binding energy with human receptors, inflammatory signal molecules, and other proteins such as human IL1R (1itb), TNFR1 (1ncf), and TNFR2 (3alq) [123].

Nigellicine, nigellidine (indazoles), nigellimine, and nigellimine N-oxide have all been found in the seeds of *N. sativa* [39]. In addition, other compounds such as arginine, palmitic, ascorbic, stearic acids, leucine, glutamic, methionine, lysine, glycine, and phytosterols are also found. Some bioactive constituents like nigellimine have the same structure as chloroquine and hydroxychloroquine [124]. They may provide similar ionophore functions to enhance Zn entry to pneumocytes to boost host immune response (proliferation and activation of neutrophils, NK cells, macrophages, and T and B cells as well as cytokine production by the immune cells) against SARS-CoV-2 by stopping the recombinant SARS-Co-RdRp activity by the inhibition of elongation and template binding. On the other hand, thymoquinone may inhibit virus binding to ACE2 on the pneumocytes [39].

SARS-CoV-2 may use human ACE2 as an attachment target to enter the host cell [41]. The RdRp in these black seeds might also stop the spread of the virus [116]. The pharmacological effects of TQ, a major bioactive component of *N. sativa*, have been suggested in some studies for numerous disorders such as respiratory distresses [40]. Kinases, heat shock proteins, and oxidorectases are targets of thymoquinone (2-methyl-5-propane-2-ylcyclohexa-2, 5-diene-1, 4-dione) and had a combined affinity with 6LU7, ACE2, and heat shock protein A5 active sites [125]. It has been reported that thymoquinone can show chemosensitizer and apoptotic activity through the downregulation of the PI3K/Akt/mTOR activation, in which an overexpression of PI3K/Akt/mTOR can be seen in patients infected with SARS-CoV-2 [126].

This medicinal plant also possesses thymol, dithymoquinone, thymohydroquinone (THQ), p-cymene, 4-terpineol, and t-anethole diagnosed by molecular docking that can inhibit COVID-19 infection [127]. *Nigella sativa* can be used prophylactically, as mentioned in Ayurvedic/Unani medicine [128]. Thymohydroquinone is one of the components of the black seed which can show different properties such as regulation of blood pressure, negative regulation of cell death, oxidative stress, regulation of immune response, and positive regulation of kinase activity [110]. Thymohydroquinone showed moderate docking energy with SARS-CoV-2 6LU7, endoribonucleoase, ADP-ribose-1ʺ-phosphatase, RNA-dependent RNA polymerase, the binding domain of the SARS-CoV-2 spike protein, and human ACE2 [129].

The anti-inflammatory activity of *N. sativa* is due to the inhibition of oxidative products of arachidonic acid called thromboxane B2, and leukotriene through blocking the activity of cyclooxygenase and lipoxygenase enzymes [36]. Thus, it is essential to manage the overexpression of cytokines in patients infected with SARS-CoV-2. Increasing the expression of CD-T cells and interferon (INF) gamma by black seed oil has been demonstrated to reduce certain viral loads in some studies [112]. *N. sativa* presents beneficial antioxidant activities through bioactive ingredients including thymoquinone, t-anethole, and 4-terpineol, and carvacrol in human preadipocytes and pretreatment of some retinal epithelial cells may be damaged by oxidative stresses [130].

The anticoagulant activity of thymoquinone of *N. sativa* has been tested, and it has been shown that this ingredient modifies cancer-associated thrombosis (CAT) and temporarily prolongs the coagulation time of thrombin time (TT), prothrombin time (PT), and activated partial thromboplastin time (aPTT) in vitro. Also, these herbal seeds are potent bronchodilators [131]. To treat viral infections, dithymoquinone (DTQ), also called nigellone, is a polymer of the carbonyl thymoquinone class that disrupts the SARS-CoV-2: ACE2 interface and the host's recognition of the virus while also affecting the S-protein pathway [132]. Black seeds are employed in pharmaceutical derivatives because of the high absorption through the stomach, high solubility, and favorable drug-likeness profile of DTQ. However, it needs to be subjected to in vitro and in vivo validation to confirm the inhibitory potency [133, 134]. In a study, it has proven that the level of inflammatory cytokine IL-8 increased after treatment by *N. sativa* extract in HeLa cells as well as downregulation of transient receptor potentials (TRP) genes such as TRPA1, TRPC4, TRPM6, TRPM7, TRPM8, and TRPV4 genes, causing decreasing viral load of coronavirus in infected cells [135, 136].

### 2.8. *Ocimum sanctum* (Tulsi)

As a family member, Lamiaceae is known as holy basil or tulsi. *Ocimum sanctum* is a fragrant perennial plant. Scientific research defines its beneficial effects. Essential oils like eugenol, extracted from tulsi leaves, have been shown to have antiviral properties [137]. *O. sanctum* is a sanctified herb mentioned in scriptures such as Ayurvedic for its medicinal features, including immunomodulatory, anti-inflammatory, antimicrobial, adaptogenic, cardioprotective, antifungal, antiviral, antibacterial, analgesic, anticancer, antiemetic, antidiabetic, antispasmodic, hepatoprotective, antifertility, and diaphoretic properties. This medicinal herb possesses dihydrodieuginol B and tulsinol A, B, C, D, E, F, and G that can inhibit the main protease and papain-like protease of SARS coronavirus [47]. Like other medicinal herbs, *O. sanctum* possesses phytochemicals diagnosed by molecular docking, which can bind with M^pro^ of novel coronavirus. SARS-CoV-2 M^pro^ can be considered a promising target in virus replication inhibition. M^pro^ is the main stimulator in viruses to produce functional proteins such as endoribonuclease, exoribonuclease, and RNA polymerase, which impede hosts intrinsic immune system function [46]. Tulsi can be used safely against SARS-CoV-2 since it has no side effects.

### 2.9. *Scutellaria baicalensis* Georgi

Baicalein is the primary active ingredient of *Scutellaria baicalensis Georgi*, a medicinal plant with anti-inflammatory and antiviral properties [138]. Research demonstrated that angiotensin-converting enzyme 2 (ACE2) and coronavirus 3CL Mpro on host epithelial cells impacted by its S-protein are the key targets for inhibiting coronavirus proliferation. At the same time, a virus-induced cytokine storm is the leading cause of consequences such as inflammation, septic shock, and multiple organ failure [139]. Baicalin had been confirmed to inhibit SARS-CoV*in vitro*, and scutellarin could interact with ACE2. Molecular docking and network pharmacology are the mainstays of pharmacological research for the treatment of COVID-19 [140]. ACE2 and 2019-nCoV-Mpro bind to baicalein and oroxylin A, indicating that they may directly affect the virus and host cells. Hence, this prevents virus proliferation, avoids the body's immunity, and blocks virus attacks. Naringenin and beta-sitosterol can regulate the expression of critical genes (CCL2, IL-1*β*, and IL-6) in the treatment of COVID-19 and produce anti-inflammatory and immune-enhancing effects through IL-17, TNF, AGE-RAGE signaling pathways, and cytokine-cytokine receptor interaction pathways [141, 142].

Anti-inflammatory actions are expected to be the main focus of SB compounds' therapeutic benefits on COVID-19 since they reduce cytokine storms and prevent the synthesis of proinflammatory cytokines [143]. TCM medicines are now the primary therapy for the COVID-19 study. In addition, Lianhua Qingwen can regulate the imbalance of ACE-Ang-II and ACE2-Ang-, leading to overwhelming proinflammatory cytokines with cytokine storm [144]. Additionally, the immunological system (MAPK, NF-B, PI3K-AKT) is regulated to prevent organ damage [145]. TCM has “multicomponent, multitarget, and multipathway” features on COVID-19. Some countries authorized chloroquine and hydroxychloroquine to treat COVID-19 [146]. However, side effects such as diarrhea and nausea might occur, making TCM therapy a must.

### 2.10. *Allium sativum* (Garlic)

As a bulbous, herbaceous plant, garlic (*Allium sativum* L.) is one of the oldest cultivated plants [147]. There are a wide variety of garlic products on the market, ranging from extracts to capsules to essential oils [148]. Garlic has been utilized medicinally and culinarily for thousands of years [149]. Human ailments have also benefited from their therapeutic effects. Anti-inflammatory, immunomodulatory, immunostimulatory, cardioprotective, hypoglycemic, antioxidant, antibiotic, antifungal, antibacterial, antiseptic, anticancer, and antiviral activities of this old medication are among its many therapeutic properties [150]. Recent studies have found alliin, allyl thiosulfinate, and s-allyl cysteine (SAC) interesting possibilities for boosting the immune system. [151]. Natural killer cells (NK cells), macrophages, lymphocytes, eosinophils, and dendritic cells (DCs) are among the most impressive immune system boosters thanks to garlic's ability to modulate cytokine production, immunoglobulin synthesis, phagocytosis, and macrophage activation [152]. After short-term treatment with the garlic extract, there are considerable increases in T lymphocytes, notably CD4+ and CD8 + T cells [153]. Patients with SARS-CoV-2 infection have been found to have decreased levels of these immunological markers, which have been linked to death in nearly all cases [153–155].

Garlic's antiviral and immunomodulatory properties have been demonstrated in clinical trials for viral cold and flu, acute respiratory viral infections, and recalcitrant multiple common warts (RMCWs) [156]. In addition, preclinical data showed that garlic and its organosulfur compounds (OSCs) have a potential antiviral activity against various human, animal, and plant pathogenic viruses by blocking viral entry into the host cells, inhibiting viral RNA polymerase, reverse transcriptase, DNA synthesis, and transcription of the immediate-early gene 1 (IEG1) and reducing the ERK/mitogen activated protease activity [157].

## 3. Future Perspectives

Patients infected with SARS-CoV-2 can hardly be treated with synthetic medications; thus, herbal remedies that possess important properties such as anti-inflammatory, antiviral, antioxidant, and similar have emerged as a viable alternative. Plant-based medicines that have been investigated for safety and efficacy and are widely available to patients due to the worldwide burden of COVID-19 can be on the frontlines of combating the ongoing tragedy caused by COVID-19. To employ bioactive secondary metabolites as medication, the issues of solubility, stability, and bioavailability must be addressed [158, 159]. For this purpose, the most effective medicinal plants were investigated to achieve the desired results and appropriate treatment as quickly as possible. But besides all these, a different way to learn about the potency of these second-metabolites is to use artificial intelligence techniques like molecular docking studies, toxicology analyses, and pharmaceutical investigations [114]. However, the mutations that occur in the targets should be considered. New medicine research tactics based on plant extracts are urgently needed to protect humans on our planet against pandemics like COVID-19, both now and in the future. Indeed, using herbs with high efficiency such as garlic, *Zingiber*, and curcuma will led us to develop more efficient antiviral therapies based on their effective components.

## 4. Conclusion

Medicinal plants can offer a viable platform for searching for medication prospects to be tested against COVID-19. The secondary metabolism of several plants functions as a treasure of phytochemicals, which have shown potential in the combat against human viruses. These herbal medicines might have the capabilities to control the synthesis and release of proinflammatory cytokines, interfere with the virus's development in host cells, and alter some RAA-related molecular pathways. Medicinal plants might be beneficial as treatments to eliminate COVID-19. Hence, it is not recommended for patients to use supplements containing one of these compounds to prevent COVID-19 or to heal the disease without particular advice or under the direct guidance of a medical professional. A suggestion for the clinician is that the management of these medicinal plants must be offered carefully to the patients, even if they are healthy. There has been a lot of contradicting information regarding these plants. Consequently, there is a risk that these therapies are related to the induction of undesirable side effects. Furthermore, preclinical and clinical trial tests of these herbal agents for COVID-19 have not been done, so more research is required.

## Figures and Tables

**Table 1:** Review of antiviral medication candidates from natural products.

Herbs	Substances	Effects	References
*Salvia miltiorrhiza*	(i) Flavonoid (e.g. tanshinone)	(i) PL^pro^ inhibitor	[23]
		(ii) 3CL^pro^ inhibitor	[24]
		(iii) Inhibition of spike protein and IS-spike protein	[25]
		(iv) Anti-inflammatory	
		(v) Cardiovascular effects	
		(vi) Antitumor	
		(vii) Increase activities of SOD (superoxide dismutase), catalase (CAT), and GSH-Px in serum	

*Glycyrrhiza glabra*	(ii) Saponin (glycyrrhizic acid)	(i) Antiviral: 1. cytoplasmic: inhibited virus replication (by production of nitrous oxide), 2. membrane: adsorption and penetration of the virus into cells by spike protein (SARS-CoV-2 S-RBD/ACE2 interaction inhibitor)	[26]
		(ii) Anti-inflammatory (modulates via an action on MAPK and toll-like receptors signaling pathways)	[27]
		(iii) Antioxidative	[28]
		(iv) Antiallergenic	[29]
		(v) Antimicrobial	[30]
		(vi) Antiparasite	
		(vii) Anticancer	

Honey	(i) Hydrogen peroxide (H_2_O_2_)	(i) Antimicrobial	[31]
	(ii) Phenolic acids (e.g., gallic acid, chlorogenic acid, syringic acid, vanillic acid, p-coumaric acid, p-hydroxybenzoic acid, and caffeic acid)	(ii) Antioxidant	[32]
	(iii) Flavonoids (e.g., quercetin, qpigenin, luteolin, chrysin, kaempferol, galangin, genistein, pinocembrin, and pinobanksin)	(iii) Anti-inflammatory	[33]
	(iv) Lysozyme	(iv) Antibacterial	[34]
	(v) 10-hydroxy-2-decenoic acid (10-HAD)	(v) Antimutagenic	
	(vi) Phenols	(vi) Antidiabetic	
	(vii) Carotenoids	(vii) Antifungal	
	(viii) Terpenes	(viii) Antitumoral	
		(ix) Antiviral	
		(x) Expedite wound healings	
		(xi) Immune booster	
		(xii) Elevates nitric oxide (NO)	
		(xiii) Cardioprotective	
		(xiv) Neuroprotective	
		(xv) 3CL^pro^ inhibitor	
		(xvi) PL^pro^ inhibitor	

*Nigella sativa*	(i) Vitamins (niacin, thiamine, riboflavin, folic acid, pyridoxine, and vitamin E)	(i) Antiviral	[35]
	(ii) Minerals (magnesium, Potassium, phosphorus, sodium, copper, calcium, and iron)	(ii) Antihypertensive	[36]
	(iii) Terpenes (e.g., thymoquinone (TQ), dithymoquinone (DTQ)	(iii) Liver tonics	[37]
	(iv) Flavanoids	(iv) Diuretics	[38]
	(v) Phytosterols	(v) Digestive	[39]
	(vi) Tannins	(vi) Antidiarrhoeal	[40]
	(vii) Coumarins	(vii) Appetite stimulant	[41]
	(viii) Phenolic compounds	(viii) Analgesics	
	(ix) Alkaloids (e.g., igellidine)	(ix) Antibacterial	
	(x) Cardiac glycosides	(x) Antioxidant	
	(xi) Saponins (e.g., a-hederin, melanin)	(xi) Anti-inflammatory	
	(xii) Unsaturated fatty acids	(xii) Immunomodulatory	
	(xiii) Volatile oils	(xiii) Bronchodilatory	
	(xiv) Proteins	(xiv) Antihistaminic	
	(xv) Essential oil	(xv) Antitussive	
		(xvi) Antitumor	

*Vitis vinifera*	(i) Resveratrol	(i) Hepatoprotective	[42]
		(ii) Cardioprotective	[43]
		(iii) Neuroprotective	
		(iv) Anti-inflammatory	
		(v) Antimicrobial	

*Zingiber*	(i) Polyphenols (e.g., diarylheptanoids)	(i) Immune enhancer	[44]
		(ii) ACE2-Ang-(1–7)-Mas pathway activater	

*Curcuma*	(i) Polyphenols (e.g., diarylheptanoids)	(i) PL^pro^ inhibitor	[45]
		(ii) Anti-inflammatory	
		(iii) Antihyperlipidemic	
		(iv) Antimicrobial	

*Ocimum sanctum* (tulsi)	(i) Dihydrodieuginol B	(i) Inhibiting virus replication (M^pro^, PL^pro^)	[46]
	(ii) Tulsinol A, B, C, D, E, F, G	(ii) Antiviral	[47]
		(iii) Adaptogenic	
		(iv) Immunomodulatory	
		(v) Antimicrobial	
		(vi) Cardioprotective	
		(vii) Anti-inflammatory	
		(viii) Antiviral	
		(ix) Antifungal	
		(x) Antibacterial	
		(xi) Antidiabetic	
		(xii) Analgesic	
		(xiii) Antifertility	
		(xiv) Anticancer	
		(xv) Antispasmodic	
		(xvi) Antiemetic	
		(xvii) Diaphoretic	
		(xviii) Hepatoprotective	

## Data Availability

All data and analysis results used for the findings of this study are available on request from the corresponding author.
